# American Dental Association

**Published:** 1876-02

**Authors:** J. H. McQillen

**Affiliations:** Cor. Sec.


### AMERICAN DENTAL ASSOCIATION.

The next meeting of the American Dental Association will be held in Philadelphia, Tuesday, August 1st, 1876. It is the desire of the officers and members of the Association that the state and local dental societies and the dental colleges of the Union, north, south, east and west, should be fully represented at that meeting, the centennial year of our national existence. This is one not only calculated to bring about that united brotherhood which we desire as fellow countrymen, but in addition as professional men the representative character of the organization makes it the most powerful and effective means for the elevation of our profession in advancing the cause of education. Speaking not for any particular section or any single interest, but for the profession at large, its recommendations assume a force that no local organization can possibly command. At the time of its inception, in 1859, there were present at the meeting at Niagra, representatives from ten local organizations. In the brief period of sixteen years that has elapsed since than, the number of state and local societies that have been formed and sending delegates to the Association has been increased seven fold. There are however, several states in which dental societies have not yet been established, it is to be hoped that the profession there will at once move in this matter and organize state and local societies and send delegates to this meeting. The representative basis of the Association demands that none other than those who come with credentials as delegates from state and local societies and dental colleges can be received as members. This however does not debar any respectable practitioner, as it is only necessary to unite with a local organization to be sent as a delegate.

The Centennial Exposition taking place in 1876 is calculated to attract among others from abroad, a number ef our professional brethren, and it is to be hoped that they will make arrangements to visit this country at the time of the meeting of the American Dental Association. If they should do so in sufficient numbers and participate in the deliberations on that occasion, the Association would assume the character of an International Congress of the profession, a consummation most earnestly to be desired. Invitations have been extended to the profession in foreign countries to unite with us on that occasion. While we should be most happy to receive delegates from foreign dental societies, reputable foreign practitioners who may present themselves without the credentials of a dental society, would be gladly welcomed and exception of the organic law would probably be made in their favor. J. H. McQillen, Cor. Sec.

